# Efficacy and safety of donepezil in patients with dementia with Lewy bodies: results from a 12‐week multicentre, randomised, double‐blind, and placebo‐controlled phase IV study

**DOI:** 10.1111/psyg.13091

**Published:** 2024-03-04

**Authors:** Etsuro Mori, Manabu Ikeda, Eizo Iseki, Sadao Katayama, Yasuhiro Nagahama, Megumi Ohdake, Takao Takase

**Affiliations:** ^1^ Department of Behavioral Neurology and Neuropsychiatry Osaka University United Graduate School of Child Development Osaka Japan; ^2^ Department of Psychiatry Osaka University Graduate School of Medicine Osaka Japan; ^3^ Senior Mental Clinic Nihonbashi Ningyocho Tokyo Japan; ^4^ Katayama Medical Clinic Okayama Japan; ^5^ Kawasaki Memorial Hospital Kanagawa Japan; ^6^ Clinical Planning and Development Department Medical HQs, Eisai Co. Ltd Tokyo Japan; ^7^ Clinical Data Science Department Medicine Development Center, Eisai Co. Ltd Tokyo Japan

**Keywords:** cholinesterase inhibitors, clinical trials, cognitive function, Lewy body dementia, phase IV, randomised controlled trials

## Abstract

**Background:**

Donepezil has been approved in Japan for the treatment of dementia with Lewy bodies (DLB) based on clinical trials showing its beneficial effects on cognitive impairment. This phase IV study evaluated the efficacy of donepezil by focusing on global clinical status during a 12‐week double‐blind phase.

**Methods:**

Patients with probable DLB were randomly assigned to the placebo (*n* = 79) or 10 mg donepezil (*n* = 81) groups. The primary endpoint was changes in global clinical status, assessed using the Clinician's Interview‐Based Impression of Change plus Caregiver Input (CIBIC‐plus). We also assessed four CIBIC‐plus domains (general condition, cognitive function, behaviour, and activities of daily living) and changes in cognitive impairment and behavioural and neuropsychiatric symptoms measured using the Mini‐Mental State Examination (MMSE) and the Neuropsychiatric Inventory (NPI), respectively.

**Results:**

Although donepezil's superiority was not shown in the global clinical status, a significant favourable effect was detected in the cognitive domain (*P* = 0.006). MMSE scores improved in the donepezil group after adjustments in *post hoc* analysis (MMSE mean difference, 1.4 (95% confidence interval (CI), 0.42–2.30), *P* = 0.004). Improvements in NPIs were similar between the groups (NPI‐2: −0.2 (95% CI, −1.48 to 1.01), *P* = 0.710; NPI‐10: 0.1 (95% CI, −3.28 to 3.55), *P* = 0.937).

**Conclusion:**

The results support the observation that the efficacy of 10 mg donepezil in improving cognitive function is clinically meaningful in DLB patients. The evaluation of global clinical status might be affected by mild to moderate DLB patients enrolled in this study. No new safety concerns were detected.

## INTRODUCTION

Dementia with Lewy bodies (DLB) is the second most common cause of degenerative dementia after Alzheimer's disease (AD).^1^ Previous studies, including clinical trials, a systematic review, and a meta‐analysis, have shown that cholinesterase inhibitors (ChEIs) improve cognitive function and neuropsychiatric symptoms of DLB.^2^, ^3^, ^4^, ^5^, ^6^, ^7^ This is due to the fact that DLB patients exhibit severe deficits in cortical levels of the neurotransmitter acetylcholine,^8^ and cholinergic neurotransmission is more impaired in DLB patients than in AD patients,^9^ although postsynaptic muscarinic receptors in the cortex are preserved in DLB patients.^10^

Donepezil (Aricept®, Eisai Co. Ltd., Tokyo, Japan), a type of ChEIs, is the world's first drug approved for DLB, and was approved in September 2014 in Japan. Prior to its approval, phase II and III clinical trials on donepezil evaluated its efficacy and safety in DLB patients. The major findings of the phase II trial, which consisted of a 12‐week double‐blind randomised control phase followed by a 52‐week open‐label extension phase, were the significant improvements on cognitive impairment measured using the Mini‐Mental State Examination (MMSE), behavioural and neuropsychiatric symptoms measured using the Neuropsychiatric Inventory (NPI), and global clinical status measured using Clinician's Interview‐Based Impression of Change plus Caregiver Input Version (CIBIC‐plus) in patients administered 5 or 10 mg donepezil. Significant improvements on CIBIC‐plus were also found at administration of 3 mg donepezil. The improvements on MMSE and NPI scores persisted for 52 weeks.^5^, ^11^

The phase III trial, which consisted of a 12‐week double‐blind confirmatory phase followed by a 52‐week open‐label extension phase, also found significant improvements in MMSE at 10 mg of donepezil.^6^, ^12^ The improvements persisted up to 52 weeks without any increased risk of major safety events, such as parkinsonism and cardiovascular disease. Although NPI scores improved at administration of 5 or 10 mg donepezil, the scores in the placebo group also improved; therefore, the trial did not find superiority of donepezil over placebo on behavioural and neuropsychiatric symptoms.

Guidelines for the clinical evaluation of antidementia drugs in Europe, USA, and Japan mutually stipulate, and to gain the antidementia indication, a drug must (i) exert its effect on the core clinical feature and (ii) have a clinically meaningful effect.^13^, ^14^, ^15^, ^16^ Based on the results of phase II and III clinical trials, donepezil was approved for treating cognitive impairment in DLB patients, contingent upon post‐marketing clinical studies evaluating its efficacy on global clinical status.

In this phase IV study, we further evaluated efficacy and safety of donepezil during a 12‐week double‐blind phase, focusing on the global clinical status. Objectives of this study were: (i) to assess superiority of donepezil over placebo in DLB patients on global clinical status (a primary endpoint); (ii) to evaluate its efficacy on cognitive impairment and behavioural and neuropsychiatric symptoms (secondary endpoints); and (iii) to evaluate safety. This study was registered at the US National Library of Medicine (NCT02345213, https://clinicaltrials.gov/ct2/show/NCT02345213) and Japan Registry of Clinical Trials (jRCT1080222744, https://jrct.niph.go.jp/en-latest-detail/jRCT1080222744).

## METHODS

### Patients

Patients diagnosed as having probable DLB according to the third consensus diagnostic criteria published in 2005^17^ were recruited from 54 participating institutions in Japan from March 2015 to January 2017. According to the first consensus diagnostic criteria published in 1996 by the DLB Consortium, patients are diagnosed as having probable DLB when they exhibit two of three clinical features (cognitive fluctuation, visual hallucinations, and parkinsonism).^18^ However, according to the 2005 updated criteria, which incorporate additional features of DLB pathology, DLB is diagnosed when patients show at least one of the three core clinical features and one of the three suggestive features (rapid eye movement sleep behaviour disorder, severe antipsychotic sensitivity, or reduced dopamine transporter uptake in basal ganglia).^17^ The criteria were revised again in 2017,^19^ but the present study used the 2005 criteria because they were in effect at the time of this study.

Inclusion criteria included age ≥50, outpatients, Clinical Dementia Rating [CDR] ≥ 0.5,^20^ MMSE score between 10 and 26,^21^ and NPI‐2 (hallucinations and cognitive fluctuation) ≥2.^5^ The caregivers routinely stayed with eligible patients at least 4 h a day and 3 days a week and agreed to provide information, assist with the treatment compliance, and escort the patients to required hospital visits during this study.

Exclusion criteria included: Parkinson's disease diagnosed at least 1 year prior to the dementia onset; use of ChEIs, central anticholinergics, and antipsychotics; focal vascular lesions (which might cause cognitive impairment) visualised on magnetic resonance imaging or computed tomographic scans; other neurological or psychiatric diseases; clinically significant systemic disease; complications or a history of severe gastrointestinal ulcer; severe asthma or obstructive pulmonary disease; hypersensitivity to donepezil or piperidine derivatives; treatment with ChEIs or any investigational drugs within 12 weeks prior to screening; severe parkinsonism (Hoehn and Yahr stage ≥ IV)^22^; systolic hypotension (systolic blood pressure < 90 mmHg); bradycardia (<50 m^−1^); sick sinus syndrome; atrial or atrioventricular conduction block; and QT interval prolongation (≥450 ms).

### Study design

**Figure 1:**
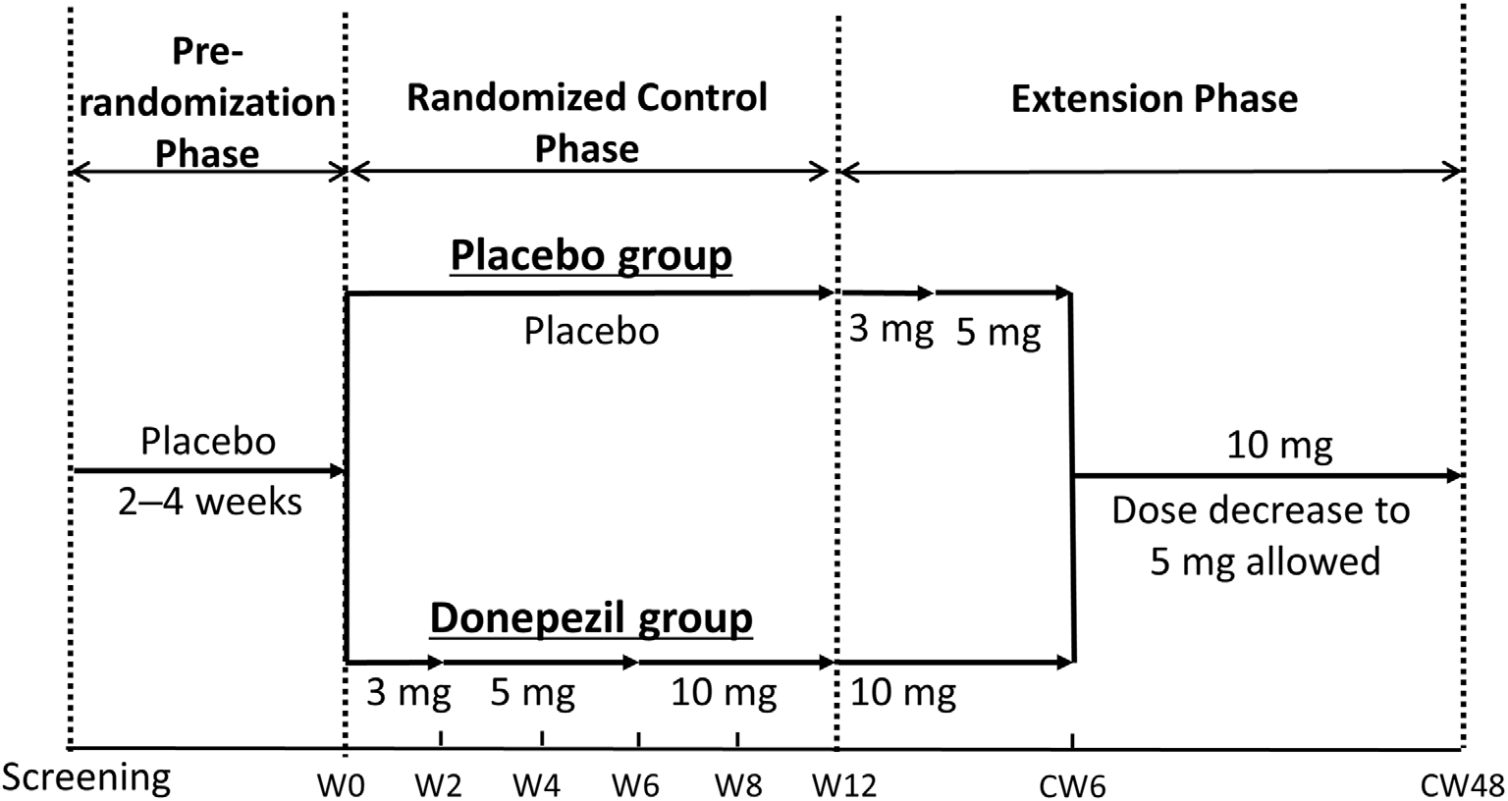
Study flow. CW, continuation week; W, week.

The phase IV study was a multicentre and placebo‐controlled study conducted in Japan and consisted of two phases: (i) a 12‐week double‐blind randomised control phase (randomised control phase) with a pre‐randomisation phase (2–4 weeks) following screening (pre‐randomisation phase); and (ii) a subsequent 48‐week open‐label extension phase (Fig. 1). In this study, we reported the results from the randomised control phase.

### Randomisation, blinding, and administration schedule

All patients were given placebo tablets during the pre‐randomisation phase for 2–4 weeks. The randomisation was performed centrally using a dynamic allocation strategy, which was adjusted according to MMSE score (≤17 or ≥18) at week 0 (baseline), and institution. Patients were assigned to placebo or donepezil in a 1:1 ratio. During the randomised control phase, the treatment assignment was blinded to all participants, including enrolled patients, their caregivers, participating physicians, nurses, and institution staff. The randomisation list was securely managed by an allocation manager.

Patients received two tablets with the same physical appearance once daily in the morning. The patients in the placebo group received two placebo tablets. For the patients in the donepezil group, the dosage was titrated at the beginning, and the treatment dosage began with 3 mg for 2 weeks (a placebo tablet and 3 mg donepezil each) and increased to 5 mg thereafter (a placebo tablet and 5 mg donepezil each) (Fig. 1). At week 6, the dosage was increased to 10 mg (two tablets of 5 mg donepezil). Dosage could be reduced to 5 mg if patients showed adverse events (AEs), in which a causal relationship with the study drug could not be ruled out (treatment‐related AEs). If dosage was reduced to 5 mg, the increase of dosage to 10 mg was not allowed. After informed consent was received, an attending physician or a participating assessor initiated disease education and caregiving advice.

### Efficacy

Prior to donepezil administration, global clinical status was assessed using Clinician's Interview‐Based Impression of Severity (CIBIS) at baseline (week 0). The primary endpoint was to assess global clinical status using CIBIC‐plus^23^ at weeks 6 and 12 in comparison with the baseline. Both CIBIS and CIBIC‐plus are semi‐structured interviews which comprehensively rate global clinical status based on the evaluation of four domains: general condition, cognitive function, behaviour, and activities of daily living. In this study, we also assessed the four CIBIC‐plus domains.

The CIBIS is rated at a seven‐point grade (1, normal; 2, borderline mentally ill; 3, mildly mentally ill; 4, moderately mentally ill; 5, markedly mentally ill; 6, severely mentally ill; and 7, most extremely ill). CIBIC‐plus is also rated at a seven‐point grade (1, marked worsening; 2, moderate worsening; 3, minimal worsening; 4, no change; 5, minimal improvement; 6, moderate improvement; and 7, marked improvement). Each CIBIC‐plus domain is rated at a 13‐point grade: the same seven‐point grade and additional six‐midpoint grade.

A trained assessor rated the global status based on observations on patients at interview and information supplied by a caregiver. CIBIS and CIBIC‐plus assessors were physicians or clinical psychologists, including experienced psychotherapists; certified individuals, such as speech therapists, occupational therapists, social workers, and psychiatric social workers; and individuals from graduate schools in psychology and social welfare, who have knowledge on dementia and its clinical symptoms and have experience in the neuropsychological examination.

Cognitive impairment was assessed with MMSE at baseline and weeks 6 and 12 (secondary endpoint).^21^ Behavioural and neuropsychiatric symptoms were assessed with NPI‐10 and NPI‐2 at baseline and week 12 (secondary endpoint). NPI‐10 comprises several subitems, including delusions, hallucinations, agitation/aggression, dysphoria, anxiety, euphoria, apathy, disinhibition, irritability/lability, and aberrant motor behaviour,^24^, ^25^ and NPI‐2 includes subitems of hallucinations and cognitive fluctuation.^5^ Each subitem of NPI is scored separately based on frequency and severity, and each score ranges 0–12. Total score of NPI‐10 or NPI‐2 is calculated by adding two or 10 scores from each subitem.

### Safety

Safety was assessed based on AEs, treatment‐related AEs, vital signs, electrocardiogram, and laboratory tests. AEs were collected and classified in accordance with Medical Dictionary for Regulatory Activities (version 20.0).

### Ethical considerations

Written informed consent was obtained from each enrolled patient (if possible) or his/her primary family member before the study. The study was conducted in accordance with the Declaration of Helsinki and Good Clinical Practice (GCP) and Good Post‐marketing Study Practice (GPSP). The protocol was approved by the institutional review board at each participating institution.

### Statistical analysis

The sample size was conservatively estimated based on the CIBIC‐plus results obtained from the phase II trial.^5^, ^11^ The Wilcoxon – Mann‐Whitney odds^26^ of donepezil against placebo were 3.06, and the 95% confidence intervals (CI) were between 1.63 and 5.75. Given the low CI of 1.63, 54 patients and 73 patients in the treatment group were respectively required to achieve an 80% and 90% statistical power in order to detect a significant difference using the Wilcoxon rank order test with Wilcoxon – Mann‐Whitney odds of 1.85 and a two‐sided significance level of 0.05. Considering the feasibility, the sample size was predefined as 140 patients (70 patients per group).

The interim analysis was conducted by an Independent Data Monitoring Committee to assess the efficacy of the primary endpoint. The interim analysis planned with 80 patients (approximately 60% of the target number of subjects) was actually performed with 84 patients.

The demographic and baseline clinical characteristics of patients were descriptively summarised. Efficacy was assessed using the full analysis set (FAS), which included patients who administered the study drug at least once and had valid efficacy assessment data at least at one point. The CIBIC‐plus distributions of the two treatment groups at week 12 were compared using a Wilcoxon two‐sample test. For MMSE and NPIs, mean score changes from baseline to 12 weeks were compared using an analysis of covariance, where the treatment groups were treated as factors and baseline values as covariates. The last observation carried forward (LOCF) approach was used for missing data at week 12, and the data of final evaluation at week 12 were thereafter written as week 12 (LOCF). Safety was assessed in the safety analysis set. AEs and treatment‐related AEs were summarised. Correlation between CIBIC‐plus cognitive function domain at week 12 (LOCF) and MMSE score changes from baseline to week 12 (LOCF) were examined using Pearson's where both CIBIC‐plus scores 1–13 and MMSE score changes were treated *ad hoc* as continuous variables.

To maintain the overall significance level of 0.05 to both sides at the final analysis of the primary CIBIC‐plus evaluation, the significance levels were determined using the modified Haybittle‐Peto method for the interim and final analysis, which were 0.01 and 0.046, respectively. Except for the primary statistical analysis, all other tests were two‐sided with a significance level of 0.05. SAS 9.3 software (SAS Institute Inc., Cary, NC, USA) was used for all statistical analyses.

## RESULTS

### Patient disposition

**Figure 2:**
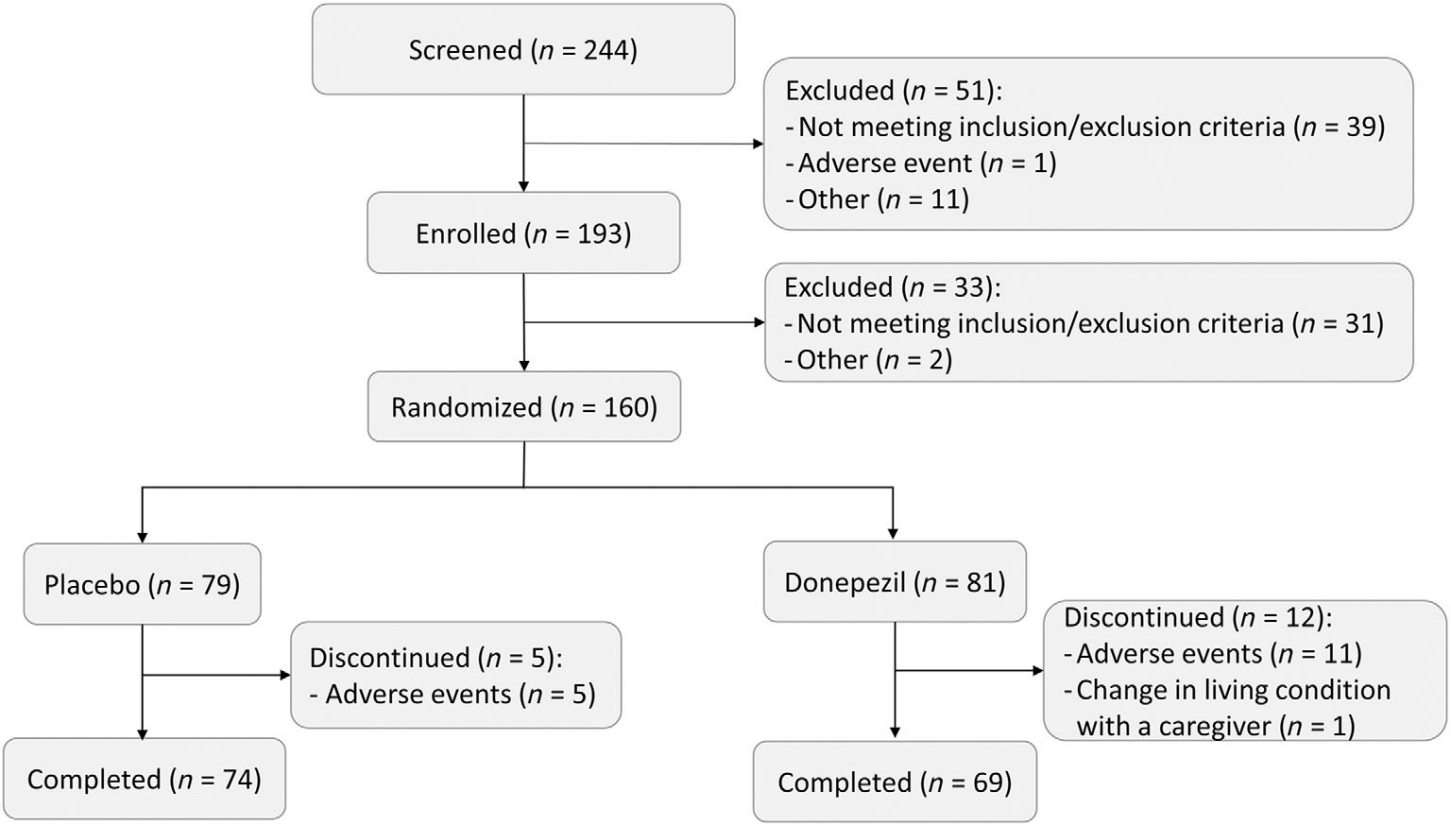
Participant disposition.

At the beginning of the pre‐randomisation phase, 244 patients were screened, and 193 patients were enrolled in the study. Among the 193 patients, 160 who were randomised to either the placebo or the donepezil group constituted the safety analysis set (79 patients in the placebo group and 81 patients in the donepezil group) (Fig. 2). Of the safety analysis set, nine patients were excluded because of no efficacy data (two patients in the placebo group and six patients in the donepezil group) or uncertain diagnosis of DLB (one patient in the placebo group). The remaining 151 patients constituted the FAS (76 patients in the placebo group and 75 patients in the donepezil group). Seventeen patients discontinued the treatment owing to AEs (five patients in the placebo group and 11 patients in the donepezil group) or a change in the living condition of a caregiver (one patient in the donepezil group).

### Patient and clinical characteristics

Table 1 shows the demographic and baseline clinical characteristics of patients in the FAS. In general, the two treatment groups had comparable distributions on three core clinical features of DLB: cognitive fluctuation (placebo: 93.4%; and donepezil: 93.3%), visual hallucinations (placebo: 67.1%; and donepezil: 65.3%), and parkinsonism (placebo: 84.2%; and donepezil: 86.7%). Major differences between the treatment groups at baseline were from: (i) CIBIS (borderline mentally ill: 2.6% in the placebo group and 10.7% in the donepezil group; moderately mentally ill: 35.5% in the placebo group and 24.0% in the donepezil group); and (ii) proportions of patients who showed MMSE scores of ≥22 (placebo: 36.8%; and donepezil: 49.3%).

**Table 1:** Demographic and baseline clinical characteristics of patients (FAS, *N* = 151)

Characteristics	Placebo (*n* = 76)	Donepezil (*n* = 75)
Sex		
Male	30 (39.5)	34 (45.3)
Female	46 (60.5)	41 (54.7)
Age, years	77.6 (7.15)	78.0 (6.44)
Weight, kg	55.17 (11.643)	52.42 (11.235)
Duration of dementia, years	2.34 (1.877)	2.15 (1.960)
History of antidementia medication	12 (15.8)	7 (9.3)
Core clinical features		
Cognitive fluctuation	71 (93.4)	70 (93.3)
Visual hallucinations	51 (67.1)	49 (65.3)
Parkinsonism	64 (84.2)	65 (86.7)
Hoehn and Yahr stage		
I	12 (15.8)	11 (14.7)
II	22 (28.9)	36 (48.0)
III	30 (39.5)	18 (24.0)
Average hours spent with the patient per day	10.9 ± 4.93	10.4 ± 4.87
CIBIS		
Normal	0 (0.0)	0 (0.0)
Borderline mentally ill	2 (2.6)	8 (10.7)
Mildly mentally ill	42 (55.3)	38 (50.7)
Moderately mentally ill	27 (35.5)	18 (24.0)
Markedly mentally ill	3 (3.9)	7 (9.3)
Severely mentally ill	2 (2.6)	4 (5.3)
Among most extremely ill	0 (0.0)	0 (0.0)
MMSE	20.0 ± 4.04	20.5 ± 4.36
NPI‐2	5.9 ± 4.27	5.7 ± 3.98
NPI‐10	12.9 ± 10.41	14.4 ± 10.90
NPI subitems		
Delusions	1.5 ± 2.68	1.3 ± 2.83
Hallucinations	2.4 ± 3.17	2.2 ± 3.08
Agitation/aggression	1.3 ± 2.25	1.4 ± 2.35
Dysphoria	1.3 ± 1.76	1.8 ± 2.65
Anxiety	1.6 ± 2.25	1.9 ± 2.69
Euphoria	0.3 ± 0.97	0.1 ± 0.51
Apathy	2.3 ± 2.82	3.0 ± 3.22
Disinhibition	0.2 ± 0.65	0.2 ± 0.78
Irritability/lability	1.0 ± 2.31	1.2 ± 2.04
Aberrant motor behaviour	1.0 ± 2.20	1.3 ± 2.80
Cognitive fluctuation	3.5 ± 2.54	3.5 ± 2.50

*Note*: Data are presented as number (%) or mean ± standard deviation (SD).

Abbreviations: FAS, full analysis set; CIBIS, Clinician's Interview‐Based Impression of Severity; MMSE, Mini‐Mental State Examination; NPI, Neuropsychiatric Inventory (NPI‐2 = hallucinations + cognitive fluctuation; NPI‐10 = delusions + hallucinations + agitation/aggression + dysphoria + anxiety + euphoria + apathy + disinhibition + irritability/lability + aberrant motor behaviour).

### CIBIC‐plus and four domains

Table 2 shows a grade distribution of CIBIC‐plus at week 12 (LOCF). Although somewhat more patients showed improvements (minimal, moderate, and marked improvement) in the donepezil group (44.6%) than in the placebo group (31.6%), the distributions of the two treatment groups were not significantly different (*P* = 0.408). For the cognitive function domain of CIBIC‐plus, more patients showed improvement (54.1%) in the donepezil group than in the placebo group (31.6%), and fewer patients showed worsening (27.0%) in the donepezil group than in the placebo group (38.2%), the difference being significant (*P* = 0.006) (Table 3). The distributions of the other three domains were not significantly different between the two treatment groups (general condition: *P* = 0.747; behaviour: *P* = 0.288; activity of daily living: *P* = 0.425) (Table 3).

**Table 2:** Distribution of CIBIC‐plus at week 12 (LOCF)

Grades	Placebo (*n* = 76)	Donepezil (*n* = 74)	*P*‐value^†^
Marked improvement	0 (0.0)	1 (1.4)	0.408
Moderate improvement	6 (7.9)	10 (13.5)	
Minimal improvement	18 (23.7)	22 (29.7)	
No change	32 (42.1)	17 (23.0)	
Minimal worsening	14 (18.4)	19 (25.7)	
Moderate worsening	5 (6.6)	5 (6.8)	
Marked worsening	1 (1.3)	0 (0.0)	

^†^ Wilcoxon two‐sample test.

*Note*: Data are presented as number (%).

Abbreviations: CIBIC‐plus, Clinician's Interview‐Based Impression of Change, plus Caregiver Input Version; LOCF, last observation carried forward.

**Table 3:** Distribution of four domains of CIBIC‐plus at week 12 (LOCF)

Domains	Grades	Placebo (*n* = 76)	Donepezil (*n* = 74)	*P*‐value^†^
1) General condition	Marked improvement	0 (0.0)	2 (2.7)	0.747
	Moderate to marked improvement	0 (0.0)	0 (0.0)	
	Moderate improvement	5 (6.6)	4 (5.4)	
	Minimal to moderate improvement	1 (1.3)	2 (2.7)	
	Minimal improvement	5 (6.6)	11 (14.9)	
	No change to minimal improvement	9 (11.8)	5 (6.8)	
	No change	39 (51.3)	25 (33.8)	
	Minimal worsening to no change	11 (14.5)	10 (13.5)	
	Minimal worsening	1 (1.3)	9 (12.2)	
	Moderate to minimal worsening	1 (1.3)	3 (4.1)	
	Moderate worsening	3 (3.9)	3 (4.1)	
	Marked to moderate worsening	0 (0.0)	0 (0.0)	
	Marked worsening	1 (1.3)	0 (0.0)	
2) Cognitive function	Marked improvement	0 (0.0)	2 (2.7)	0.006
	Moderate to marked improvement	0 (0.0)	0 (0.0)	
	Moderate improvement	3 (3.9)	6 (8.1)	
	Minimal to moderate improvement	1 (1.3)	2 (2.7)	
	Minimal improvement	10 (13.2)	15 (20.3)	
	No change to minimal improvement	10 (13.2)	15 (20.3)	
	No change	23 (30.3)	14 (18.9)	
	Minimal worsening to no change	6 (7.9)	8 (10.8)	
	Minimal worsening	18 (23.7)	10 (13.5)	
	Moderate to minimal worsening	2 (2.6)	1 (1.4)	
	Moderate worsening	3 (3.9)	0 (0.0)	
	Marked to moderate worsening	0 (0.0)	0 (0.0)	
	Marked worsening	0 (0.0)	1 (1.4)	
3) Behaviour	Marked improvement	0 (0.0)	2 (2.7)	0.288
	Moderate to marked improvement	1 (1.3)	1 (1.4)	
	Moderate improvement	8 (10.5)	8 (10.8)	
	Minimal to moderate improvement	1 (1.3)	3 (4.1)	
	Minimal improvement	11 (14.5)	13 (17.6)	
	No change to minimal improvement	10 (13.2)	12 (16.2)	
	No change	26 (34.2)	19 (25.7)	
	Minimal worsening to no change	10 (13.2)	4 (5.4)	
	Minimal worsening	6 (7.9)	5 (6.8)	
	Moderate to minimal worsening	0 (0.0)	2 (2.7)	
	Moderate worsening	2 (2.6)	3 (4.1)	
	Marked to moderate worsening	0 (0.0)	1 (1.4)	
	Marked worsening	1 (1.3)	1 (1.4)	
4) Activities of daily living	Marked improvement	0 (0.0)	1 (1.4)	0.425
	Moderate to marked improvement	0 (0.0)	0 (0.0)	
	Moderate improvement	3 (3.9)	1 (1.4)	
	Minimal to moderate improvement	1 (1.3)	0 (0.0)	
	Minimal improvement	5 (6.6)	7 (9.5)	
	No change to minimal improvement	8 (10.5)	7 (9.5)	
	No change	43 (56.6)	36 (48.6)	
	Minimal worsening to no change	8 (10.5)	10 (13.5)	
	Minimal worsening	3 (3.9)	9 (12.2)	
	Moderate to minimal worsening	2 (2.6)	2 (2.7)	
	Moderate worsening	2 (2.6)	1 (1.4)	
	Marked to moderate worsening	0 (0.0)	0 (0.0)	
	Marked worsening	1 (1.3)	0 (0.0)	

^†^ Wilcoxon two‐sample test.

*Note*: Data are presented as number (%).

Abbreviations: CIBIC‐plus, Clinician's Interview‐Based Impression of Change, plus Caregiver Input Version; LOCF, last observation carried forward.

### Cognitive impairment

The mean MMSE score changes from baseline to week 12 (LOCF) were similar in both groups (placebo: 0.7 ± 0.35; donepezil: 1.6 ± 0.35), with the between‐group mean difference of 0.9 (95% CI, −0.08 to 1.86; *P* = 0.072) (Table 4). However, in *post hoc* analysis, from screening to baseline, the mean MMSE score of the placebo group declined (−0.1 ± 2.55), but that of the donepezil group increased (1.0 ± 2.45) (two‐sample *t*‐test, *P* = 0.006). Because of the unexpectedly uneven changes from screening to baseline, a statistical analysis was adjusted with these score changes, and the adjusted mean difference in MMSE scores from baseline to week 12 (LOCF) was significant between the donepezil group (1.8 ± 0.33) and the placebo group (0.5 ± 0.33), with the between‐group mean difference of 1.4 (95% CI, 0.42–2.30; *P* = 0.004). The CIBIC‐plus cognitive function domain score was correlated with MMSE score changes from baseline to week 12 (Pearson's correlation coefficient = 0.576).

**Table 4:** Mean MMSE scores and mean differences from baseline to week 12 (LOCF)

Parameter	Visit	*n*	Placebo (*n* = 76)	*n*	Donepezil (*n* = 75)	Least square mean difference (95% CI)	*P*‐value
MMSE	Screening (SD)	75	20.1 ± 3.89	75	19.5 ± 4.63	‐	‐
	Baseline (SD)	76	20.0 ± 4.04	75	20.5 ± 4.36	‐	‐
	Mean difference from screening to baseline (SD)	75	−0.1 ± 2.55	75	1.0 ± 2.45	‐	0.006^†^
	Week 12 (LOCF) (SD)	76	20.8 ± 4.81	74	22.1 ± 4.75	‐	‐
	Least square mean difference from baseline to week 12 (LOCF) (SE)	76	0.7 ± 0.35	74	1.6 ± 0.35	0.9 (−0.08 to 1.86)	0.072^‡^
	Least square mean difference from baseline to week 12 (LOCF) (SE), adjusted	76	0.5 ± 0.33	74	1.8 ± 0.33	1.4 (0.42 to 2.30)	0.004^§^

^†^ Two‐sample *t*‐test.

^‡^ Analysis of covariance with treatment groups as factors and baseline values as covariates.

^§^ Analysis of covariance with treatment groups as factors and baseline values and changes in values from screening to baseline as covariates.

*Note*: Data are presented as mean ± standard deviation (SD) or standard error (SE).

Abbreviations: MMSE, Mini‐Mental State Examination; LOCF, last observation carried forward; CI, confidence interval.

### Behavioural and neuropsychiatric symptoms

NPI‐2 scores declined at week 12 (LOCF) from baseline in both groups, and the mean score differences were similar in the placebo (−0.9 ± 0.44) and donepezil (−1.2 ± 0.45) groups, with a between‐group mean difference of −0.2 (95% CI, −1.48 to 1.01; *P* = 0.710) (Table 5). NPI‐10 scores also declined at week 12 (LOCF) from baseline, and the mean score differences were also comparable in both groups (placebo: −1.1 ± 1.21; donepezil: −1.0 ± 1.23), with a between‐group mean difference of 0.1 (95% CI, −3.28 to 3.55; *P* = 0.937).

**Table 5:** Mean NPI‐10 and NPI‐2 scores and mean differences from baseline to week 12 (LOCF)

Parameter	Visit	*n*	Placebo (*n* = 76)	*n*	Donepezil (*n* = 75)	Least square mean difference (95% CI)	*P*‐value^†^
NPI‐2	Screening (SD)	75	6.0 ± 4.88	75	5.4 ± 3.58	‐	‐
	Baseline (SD)	76	5.9 ± 4.27	75	5.7 ± 3.98	‐	‐
	Week 12 (LOCF) (SD)	75	4.9 ± 5.18	72	4.4 ± 4.59	‐	‐
	Least square mean difference from baseline to week 12 (LOCF) (SE)	75	−0.9 ± 0.44	72	−1.2 ± 0.45	−0.2 (−1.48 to 1.01)	0.710
NPI‐10	Screening (SD)	75	13.7 ± 12.29	75	14.3 ± 11.31	‐	‐
	Baseline (SD)	76	12.9 ± 10.41	75	14.4 ± 10.90	‐	‐
	Week 12 (LOCF) (SD)	75	11.8 ± 13.30	72	13.3 ± 14.35	‐	‐
	Least square mean difference from baseline to week 12 (LOCF) (SE)	75	−1.1 ± 1.21	72	−1.0 ± 1.23	0.1 (−3.28 to 3.55)	0.937

^†^ Analysis of covariance with treatment groups as factors and baseline values as covariates.

*Note*: Data are presented as mean ± standard deviation (SD) or standard error (SE).

Abbreviations: NPI, Neuropsychiatric Inventory; LOCF, last observation carried forward; CI, confidence interval.

### Safety

In the safety analysis set, the incidence of AEs was 43.0% in the placebo group and 69.1% in the donepezil group (Table 6). Preferred term AEs with ≥5% incidence in any treatment groups were viral upper respiratory tract infection (12.3%) and nausea (11.1%), which were both in the donepezil group. The AEs were higher in the donepezil group than in the placebo group. Serious AEs including death were reported in 10 patients: four patients in the placebo group and six patients in the donepezil group. One patient died due to serious AEs (pneumonia and atrial fibrillation) in the donepezil group, but the causal relationship with donepezil was denied.

**Table 6:** AEs and treatment‐related AEs with an incidence of ≥5% in the treatment groups (SAS, *n* = 160)

MedDRA system organ class and preferred term	Placebo (*n* = 79)		Donepezil (*n* = 81)	
	AEs	Treatment‐related AEs	AEs	Treatment‐related AEs
Total incidence	34 (43.0)	12 (15.2)	56 (69.1)	24 (29.6)
Gastrointestinal disorders	8 (10.1)	1 (1.3)	16 (19.8)	12 (14.8)
Nausea	1 (1.3)	1 (1.3)	9 (11.1)	8 (9.9)
Infections and infestations	8 (10.1)	0 (0.0)	16 (19.8)	0 (0.0)
Viral upper respiratory tract infection	3 (3.8)	0 (0.0)	10 (12.3)	0 (0.0)

*Note*: Data are presented as number (%). Data show preferred terms with an incidence of more than 5%. Treatment‐related AEs are AEs in which causal relationship with the study drug was considered possible or probable.

Abbreviations: AE, adverse events; SAS, safety analysis set.

In the safety analysis set, the incidence of treatment‐related AEs was 15.2% in the placebo group and 29.6% in the donepezil group (Table 6). Preferred term treatment‐related AEs with ≥5% incidence in any treatment groups were nausea, which was higher in the donepezil (9.9%) group than in the placebo group (1.3%). Serious treatment‐related AEs were reported in two patients in the placebo group (diminished level of consciousness or drug‐induced liver injury) and one patient in the donepezil group (parkinsonism).

Regarding vital signs and electrocardiogram, abnormal blood pressure changes (systolic blood pressure of >180 mmHg and the increase of systolic blood pressure ≥20 mmHg from baseline) were observed within 12 weeks in five patients (6.3%) in the placebo group and none (0%) in the donepezil group, and abnormal body weight changes (reduced body weight of ≥7% from baseline) were observed in five patients (6.3%) in the placebo group and 10 patients (12.3%) in the donepezil group. Clinically important abnormality in electrocardiogram at week 12 (LOCF) was observed in three patients (3.9%) in the placebo group and four patients (5.3%) in the donepezil group.

## DISCUSSION

In this phase IV study, we assessed the efficacy of donepezil in DLB patients on global clinical status using CIBIC‐plus, a primary endpoint, during the12‐week double‐blind phase. Although we failed to detect the superiority of donepezil over placebo on global clinical status, the cognitive function domain of CIBIC‐plus significantly improved in the donepezil group compared with the placebo group. Cognitive impairment assessed by MMSE also significantly improved in the donepezil group when adjusted with score changes from screening to baseline in *post hoc* analysis. Improvements in behavioural and neuropsychiatric symptoms measured by NPI‐2 and NPI‐10 were comparable in these two groups, and this study failed to confirm the findings of the phase II study in which donepezil was shown to improve the symptoms. For the safety, the incidence of AEs was 43.0% in the placebo group and 69.1% in the donepezil group, and the incidence of treatment‐related AEs was 15.2% in the placebo group and 29.6% in the donepezil group. AEs were of well‐known types and incidence, and no new safety concerns were noted in patients receiving donepezil.

### Clinically meaningful benefits of improved cognitive function

In this clinical study on donepezil, we for the first time analyzed each element of the global clinical status evaluation. The efficacy of donepezil on the cognitive function was confirmed by the CIBIC‐plus cognitive function domain score, which was comprehensively assessed by a trained third party based on not only neuropsychological tests of patients but also their conditional changes that the patients and their caregivers perceived through daily lives. This strongly indicates that significant improvements in the measured level of cognitive impairment, assessed using MMSE in patients treated with donepezil and consistently observed in this study as well as in the previous phase II and III clinical trials,^5^, ^6^ suggest clinically meaningful improvements that can be recognised in daily lives. This is supported by the fact that the CIBIC‐plus cognitive function domain score was highly correlated with MMSE score changes.

Such improvements in cognitive function also suggest the social significance of donepezil. Caregiver burden can be substantial in DLB patients compared with AD patients^5^, ^27^ partly because the central feature of DLB (progressive cognitive decline of sufficient magnitude to interfere with normal social or occupational function)^17^, ^18^ advances at a similar or faster rate in DLB patients than in AD patients.^28^, ^29^, ^30^, ^31^ The risk of a fall, which is presumably related to visuocognitive impairment and inattention, is also higher in DLB patients than in AD patients.^32^, ^33^ Injuries and fractures resulting from cognitive impairment and falls increase burden not only in DLB patients but also in their caregivers, and patients may be hospitalised or admitted to long‐term facilities at early stages, consequently increasing medical expenses. In fact, the time to hospitalisation or admission to long‐term facilities is shorter in DLB patients than in AD patients,^34^, ^35^ and the medical expenses are considerably higher in DLB patients.^36^, ^37^ Therefore, the treatments of the primary symptoms of DLB, such as cognitive impairment, are an important issue to tackle in the area of medical and long‐term care of ageing populations. Under such circumstances, the improvements in cognitive function in this study are likely to be socially significant.

### Possible reasons for insignificant improvements on CIBIC‐plus

Patients with mild to moderate DLB may have been over‐represented in this study compared with previous phase II and III clinical trials. For instance, of the three core features of DLB, visual hallucinations showed lower prevalence in this study (66.2%) than in phase II (82.2%) and phase III (87.0%) trials, although the prevalence of the other two core features was similar.^5^, ^6^ Percentages of patients who had a lower clinical dementia rating (CDR = 0.5) were higher in this study (31.8%) than in trials of phase II (28.9%)^5^ and phase III (26.1%).^6^ In addition, the mean baseline scores of NPI‐2 and NPI‐10 were lower in this study (NPI‐2: 5.8 ± 4.11; NPI‐10: 13.7 ± 10.65) than in the phase II trial (NPI‐2: 6.9 ± 4.6; NPI‐10: 18.2 ± 11.2) and phase III trial (NPI‐2: 7.1 ± 4.5; NPI‐10: 18.6 ± 14.0).^5^, ^6^ CIBIC‐plus assessments depended on the extent of patients' conditional changes; however, the changes might be difficult to capture in mild to moderate DLB patients. The patient characteristics in the study population may have affected our results on CIBIC‐plus.

The sample size for this phase IV study was conservatively set based on phase II, which are the only existing phase II data for CIBIC‐plus. However, the environment surrounding DLB patients changed since the previous phase II and III clinical trials on donepezil. Here, we need to consider several reasons why the clinical characteristics of patients in this study were different from those in the previous trials. First, this study used the third consensus diagnostic criteria published in 2005,^17^ while the previous two clinical trials used the criteria published in 1996.^18^ However, the impacts of the 2005 criteria on our results were perhaps minimal because only nine patients were additionally diagnosed with probable DLB under the 2005 revised criteria (DLB is diagnosed when one of three core features and one of three supportive features are identified in patients). Second, after donepezil was approved in Japan, increased DLB awareness has led to its widespread diagnosis, and this change probably reduces the number of missing diagnoses and leads to a detection of DLB patients at even earlier stages than before. Because of these changes, typical cases of DLB, such as those with visual hallucinations, who could be diagnosed and treated in general institutions, did not need to visit specialty institutions where clinical studies were conducted. Third, because the approved medication is already on the market, treatments are prioritised over clinical trials. Patients with severe symptoms, such as visual hallucinations, incur considerable caregiver burden, and patients with severe DLB might have been less likely to be enrolled in such clinical trials. Therefore, this phase IV study might have been underpowered because of reduced effect sizes due to differences in patients' background characteristics in the post‐marketing environment.

Even though the phase IV study was conservatively designed to verify the efficacy of donepezil under the situation where only the phase II data were available to estimate the effect size of CIBIC‐plus for DLB patients, the primary endpoint in the phase IV study did not reach statistical significance. Although almost the same population as the phase II trial was targeted, consequently it was considered that any potential differences in the characteristics of the patients' background were caused under the post‐marketing environment and might have affected CIBIC‐plus assessment, the nature of which seems to be susceptible to various factors.

## CONCLUSION

In this phase IV study, we evaluated the efficacy and safety of donepezil in DLB patients during a 12‐week double‐blind phase. Although there was no improvements in global clinical status, this study confirmed significant improvements in cognitive impairment not only on MMSE in *post hoc* analysis, which conforms to the previous phase II and III clinical trials, but also on the cognitive function domain in CIBIC‐plus assessments. This study further indicated clinically meaningful benefits of donepezil on cognitive function in patients with DLB. No new safety concerns arose in patients receiving donepezil in this study.

## FUNDING INFORMATION

The funding for this study was provided by Eisai Co., Ltd. (Tokyo, Japan).

## ETHICS STATEMENT

Written informed consent was obtained from each enrolled patient (if possible) or his/her primary family member before the study. The study was conducted in accordance with the Declaration of Helsinki and GCP and GPSP. The protocol was approved by the institutional review board at each participating institution.

## Data Availability

The data are not publicly available due to privacy or ethical restrictions.
